# Reforming birth registration law in England and Wales?

**DOI:** 10.1016/j.rbms.2017.07.001

**Published:** 2017-08-10

**Authors:** Julie McCandless

**Affiliations:** LondonSchool of Economics, London, UK

**Keywords:** birth registration, birth certificates, family law, illegitimacy, Law Commission, assisted and collaborative reproduction

## Abstract

The Law Commission of England and Wales is considering what its 13^th^ Programme of Law Reform should address. During the consultation process, a project on birth registration law has been mooted. This is a very welcome proposal given that civil birth registration in England and Wales is a compulsory procedure that not only finds its roots in the early Victorian era, but also remains very similar, at least in terms of form and the information that is recorded. I first use two recent legal challenges to illustrate why the current system is coming under increasing pressure. I further use these examples to caution against a law reform agenda that is narrowly focused on the precise information recorded, without a preliminary and wider examination of what the role and purpose of birth registration is, and should be, in society. I argue that this needs to be addressed before the state can justify the parameters of the information recorded. I then use an outline of historical reforms relating to the registration of births outside of marriage to highlight the normative two-parent family model that underpins the birth registration system. I argue that legal reform must be cognizant of the tenacity of this normative family model, particularly in relation to reform proposals surrounding donor conception and the annotation of birth certificates. Finally, I draw attention to wider developments in family law that cast birth registration as a social policy tool for the facilitation of parent–child relationships, particularly unmarried fathers.

## Introduction

The Law Commission of England and Wales is currently considering what its 13^th^ Programme of Law Reform should address. During the consultation process, a project on birth registration law and certificates has been mooted. This is a very welcome proposal. Civil birth registration in England and Wales is a compulsory procedure that not only finds its roots in the early Victorian era, but also remains very similar, at least in terms of form and the information that is recorded. In this short commentary, I first use two recent legal challenges to illustrate why the current system is coming under increasing pressure. I further use these examples to caution against a law reform agenda that is narrowly focused on the precise information recorded on a birth certificate, particularly in relation to parenthood. This is not to suggest that the information recorded should not be reconsidered, but rather that the scope of a law reform project on birth registration needs to be wider given the normative family model which underpins the system.

I then draw particular attention to the wider family law and policy context within which birth registration operates, and ask what this means in relation to the information recorded. I outline reforms relating to the registration of births outside of marriage to demonstrate that, while historical changes could be interpreted as being a bit haphazard, they have, in fact, always been informed by the normative two-parent family model. I argue that legal reform cannot ignore this tenacious normative dimension, given the extent to which it shapes how the familial information on a birth certificate is understood. I also point to changes in the Adoption and Children Act 2002 and the Welfare Reform Act 2009 – relating to the joint birth registration of births outside of marriage – to show how birth registration has been conceptualized in recent times by the state as a social policy tool that facilitates a parent–child relationship. I argue that this imbues birth registration with even further significance in terms of ‘textually mediated’ statecraft (Breckenridge and Szreter, 2012), for it means that registration is significant for purposes beyond ‘recognition’. Legal reform must be cognizant of this growing role of bureaucracy and ‘paperwork’ in the state’s efforts to organize and determine family practices and behaviour. I conclude that a law reform process underpinned by these wider considerations should be very much welcomed as a way of progressively considering what role this compulsory civil registration procedure can and should play in contemporary and future society.

## Challenging times for birth registration

It has been almost a decade since the Joint Committee scrutinizing the Human Tissues and Embryos (Draft) Bill (2007) recommended that, 'as a matter of urgency', the Government give consideration to rights that may be implicated in state authorities holding personal information – namely details of gamete donors – and the information that appears on birth certificates [House of Lords and House of Commons Committee on the Human Tissue and Embryos (Draft) Bill, 2007: 276]. No government has responded to this recommendation. This is perhaps not surprising as, while donor conception is not uncommon, it affects only a relatively small proportion of the population.^1^ However, the existence of a similar minority demographic represented by mother-only registered births (around 7% of births registered each year; Wallbank, 2009: 1) did not prevent the enactment of quite considerable reforms in relation to the compulsory joint registration of births outside of marriage in the Welfare Reform Act 2009 (Schedule 6; on the detail of the reforms, see below). However, the fact that these reforms have never been implemented points to a further – arguably more likely – reason why the Joint Committee’s recommendation has been gathering dust; that to reform the law relating to birth registration and certificates is a complex task. The interplay between public and private information is politically sensitive, and the myriad uses for a birth certificate – including passport application, genealogical research, application for school entrance and local authority services^2^ – makes it difficult to determine the most obvious government department for such an undertaking. The Law Commission, as a non-political entity, is therefore well placed to start considering how this complex area of law might be reformed.^3^

In relation to the scope of a possible law reform project, I was very pleased to see the Law Commission cast this in wider terms than the Joint Committee recommendation. Any attempt to reform this one area of birth registration law is likely to prove unsatisfactory, given that the issue of whether birth certificates should indicate that a person is donor-conceived and/or include the name(s) of the gamete provider(s) continues to prove highly controversial and often polarized. In my view, this stems not only from different values being placed on genetic relationships, but also from different understandings of the purpose of birth registration.

To say this is not to suggest that the issue should not be included in a law reform programme, particularly in light of the depth of feeling that it evokes. However, two recent legal challenges signal that a much wider review is warranted. The first of these cases appears, on the surface, to fit closely with the Joint Committee’s concerns. In 2014, Emma Cresswell had her birth certificate re-issued without the name of the man originally recorded as her father, who she had assumed was her biological father. Her legal action followed an argument whereby he disclosed that she and her two brothers had been conceived through sperm donation (Crossley, 2014, Rowley, 2014). She was 19 when this argument took place and she campaigned for almost 6 years to have her birth certificate re-issued. In media interviews, Emma Creswell has indicated that she is now part of a wider campaign group that wants birth certificates to record that a person was donor-conceived (Crossley, 2014, Rowley, 2014). However, her specific legal campaign was never going to achieve that, at least not directly.^4^ This allows us to interpret her campaign as being about a wider issue, which is the extent to which people see – and want to see – their birth certificate reflect their lived familial experience. Emma did not have a close relationship with the man recorded as her father, having had little contact with him for most of her childhood (Crossley, 2014, Rowley, 2014). While her campaign was clearly prompted by finding out that she did not share a genetic relationship with him, the context of their relationship may also have been a motivating factor. We should therefore be cautious about reading her legal challenge in a reductive way, not least because it taps into a growing dissatisfaction with the prescriptive nature of what is recorded on birth – and other – certificates in contexts whereby individuals feel that their subjective experience and lived realities are ‘inaccurately’ represented, or they seek official recognition of a life event.^5^ While donor conception and other collaborative^6^ reproductive practices such as surrogacy seem to present the most direct challenges to the purpose and form of birth registration and certificates, my second case example demonstrates that challenging encounters with the current birth registration system are much wider in scope.

In a recent High Court case, a trans woman known as JK challenged the legal requirement that she be recorded as ‘father’ on her children’s birth certificates. Despite self-identifying and living her life as a woman, in the absence of a gender recognition certificate – which takes some time to acquire (see further Grabham, 2010) – JK was legally regarded as male, as per her original birth certificate {*JK v Register General for England and Wales* [2015] EWHC 990 (Admin)}.^7^ To complicate matters further, JK’s male name was recorded on her first child’s birth certificate, while her female name appeared on her second child’s. Finally, while Emma Cresswell was conceived through clinically assisted means, JK’s children were conceived through sexual intercourse with her partner before JK commenced female hormonal treatment. JK’s case therefore highlights the wider reach of challenges to the birth registration system beyond assisted reproduction. It also highlights the challenging interplay of prescriptively recorded details, with the more fluid reality of gender identity, family connections and naming practices; or at least the difficulties presented by a record that remains fixed in time, but which must be used throughout the life-course. Birth registration also records places, occupations, dates and adult legal relationships, none of which are beyond contestation.

Any standardized written record of vital events will inevitably flatten subjectivities and the richness of an individual’s personal narrative. However, what these two cases demonstrate is that individuals are increasingly prepared to challenge the birth registration system when they regard the information recorded as ‘inaccurate’. For both Emma Cresswell and JK, this was, in part, motivated by the fact that other people see birth certificates for a wide variety of reasons, and thus have access to the information recorded. JK’s case also came about quite simply because birth registration was not straightforward for her family. While cases like JK’s may seem exceptional in the context of an administrative procedure that works for most people, this does not mean that they are unimportant, for not only do they highlight the particular difficulties and injustices for the individuals involved, but they also illuminate wider problems with law and a compulsory civil procedure such as birth registration.

Thinking about how birth registration law could and should be reformed is undoubtedly a complex task. In its consultation, the Law Commission asked a number of questions relating to the specifics of the information that is recorded on a birth certificate. These are all important questions to be addressed. However, the Commission also posed two more principled questions: What is the purpose of a birth certificate? For whose benefit is the record kept? While it would be naïve to think that we could completely future-proof how birth registration comes to be understood in society, effective legal reform must be underpinned by a principled consideration of its role and purpose in contemporary society, for only then can we determine and justify the parameters of the information that should be recorded. By addressing questions such as these, the Law Commission would be making a good start in instigating a durable – as opposed to piecemeal – law reform process of what is essentially an early Victorian system. It is to the history of birth registration and reforms relating to the registration of births outside of marriage that I now turn.

## Birth registration law and historic reform

There has been a centralized system of civil birth registration in England and Wales^8^ since 1837, following the Births and Deaths Registration Act 1836 (the 1836 Act) (see further Higgs, 2004, Probert, 2011). The Births and Deaths Registration Act 1874 (the 1874 Act) made the registration of births compulsory by placing a legal duty on certain ‘informants’ to notify the local Registrar of a birth.^9^ The current law governing birth registration is the 60-year-old Births and Deaths Registration Act 1953 (the 1953 Act), which – rather quietly – celebrated its diamond jubilee around the same time as Queen Elizabeth II. This legislation has seen little by way of reform, and in comparing my birth certificate with that of my (almost sexagenarian) mother, there is very little difference in the information recorded – my mother’s ‘usual address’ was recorded on my birth certificate, while her father’s address was recorded on hers – not least because I was born before 1986 which is when the mother’s occupation started to be recorded as well as the father’s.^10^ The main amendments to the 1953 Act relate to the incorporation of ‘female parents’ following changes enacted by the Human Fertilisation and Embryology Act 2008, and a multiplication of the documentation that can be provided by an unmarried father when he cannot attend in person to register his child’s birth with the mother (see Children Act 1989). However, other than permitting two female parents to be named on the birth certificate, the information recorded has remained fairly constant; to include a space for the mother’s ‘maiden name’ if different from her surname following civil partnership or marriage to her female partner, as has been traditional for heterosexual couples.

When viewed in this light, it is easy to see the birth registration system as rather rigid and failing to keep up with modern family life; to a large extent, this is true. However, a brief historical examination of how the system has been reformed in relation to births outside of marriage provides examples of the civil birth system being remoulded in response to changing societal mores. The introduction of civil birth registration has its roots in a local system of parish registration introduced by Thomas Cromwell (see further Szreter, 2012). Although recording the religious rites of baptism, marriage and burial, this parish system was more concerned with recording and facilitating property rights and inheritance than it was with religious doctrine (Higgs, 2004). As a consequence, the 1836 Act made no provision for the recording of a ‘putative father’, as what mattered was establishing a legitimate line of descent.^11^ However, the 1874 Act introduced provisions to record the father’s name if he attended to register the birth with the mother. In the 1953 Act, this extended to also being recorded without attending in person, so long as some form of legal paperwork – such as a statutory declaration and, later, a parental responsibility agreement or order under the Children Act 1989 – was submitted by the mother. These changes, in part, provided some sort of state recognition to an unmarried father who was willing to acknowledge his ‘illegitimate’ child (on the role of registration and recognition, see Breckenridge and Szreter, 2012). The Legitimacy Act 1926 introduced a requirement to ‘re-register’ a birth following the marriage of the child’s parents, thus ‘legitimizing’ the child in the eyes of the law.

It is difficult for persons of my generation and younger to fully appreciate the effects of an illegitimate birth and illegitimacy. However, these were clearly stigmatizing and severe, and in 1947, we arguably see an official sensitivity to these stigmatizing effects with the introduction of the ‘short’ birth certificate. These certificates record only the name, sex, date and place of birth, without disclosing any parental details. Although the utility of the short birth certificate may be questionable today, given that the long birth certificate is increasingly requested for identification purposes,^12^ the short version has probably been very useful in the past for enabling persons to keep the circumstances of their family private. However, I have a different motivation for mentioning the short birth certificate, which is to question whether the introduction of a documentary mechanism that enables individuals to keep this information private is in tension with the increasing ability of unmarried fathers to be recorded on the birth certificate? The answer may be a straightforward ‘no’, given the qualitative difference between making information available to the persons it is about, in contrast to the world at large. However, as with most things, I suspect there were other issues at play. For example, there may have been some value in having this information recorded, either for the purpose of statistical analysis or the provision of child maintenance. Or, the explanation could be something more normative, given that the registration located the child within a family unit that at least resembled the normative two-parent marital family.

This explanation seems particularly significant given that the most recent legislative reforms to the system have further facilitated the registration of unmarried fathers by removing the need for legal documentation in the case of voluntary joint birth registration with the child’s mother (Adoption and Children Act 2002, s 111), and by introducing compulsory joint birth registration in all but a narrow range of exceptional cases (Welfare Reform Act 2009, Schedule 6). I discuss this normative dimension further below. For now, I want to end this historical outline by noting that the requirement to ‘re-register’ a birth following the marriage or civil partnership of the child’s parents still forms part of the current legislative framework.^13^ This seems really odd in an era where we have abolished direct legal distinctions between ‘legitimate’ and ‘illegitimate’ children.

## Future reform?

From my brief exploration of reforms relating to the registration of births outside of marriage, I want to make four observations. The first is that where there is political will, the law in this area can of course be reformed, despite the complexity of the task. This should remove any notion that reforming what might seem for many to be an intuitive, ingrained and straightforward procedure would be overly difficult or confusing, and somehow not worth the effort, time or costs involved. The ongoing requirement to re-register an ‘illegitimate’ birth is, by itself, a clear enough signal that reform of this 'old-age pensioner' of the statute books is needed, before even starting to think through the challenges heralded by developments in assisted and collaborative reproduction.

The second observation is that historical reform of birth registration law could be interpreted as being a bit haphazard, in that different trends – such as the registration of more unmarried fathers on birth certificates and the introduction of short birth certificates – seem to occur along similar time trajectories. I mention this as I think it will be an observation that will be invoked in relation to the extent to which genetic parentage is recorded on a birth certificate. Here, there is potential for the increasing ability of unmarried (genetic) fathers to register their connection to a child to be pitched as in tension with the parenthood provisions of the Family Law Act 1987 and the Human Fertilisation and Embryology Act 1990, which made explicit provision for non-genetic parents – male or female – to be registered as legal parents, with no indication on a birth certificate that the child was conceived through donor conception. However, this seems to be only a partial telling of a story, which perhaps finds its origins in the inaccurate characterization by the Warnock Committee of the birth register 'as a true genetic record' (Warnock, 1984: 4.21). While it is fair to say that the parents recorded on a birth certificate have often been a child’s genetic parents, there is very little evidence that birth registration was ever meant to confer certainty about genetic parentage. While we may want to deliberate on whether a future system could or should confer this certainty, it would be disingenuous to start this discussion with the argument that this has always been the purpose of birth registration until legal changes heralded by assisted reproduction took place. The picture is more complex in that what the process recorded in terms of parenthood was a legally prescribed relationship, at birth, to the child who was being registered. While legal presumptions operated – and continue to operate – to confer parental status on certain persons who may have a biogenetic connection to a child, these presumptions have never guaranteed the presence of a genetic connection, particularly in relation to fatherhood.

Furthermore, in terms of birth certificates not acknowledging the role of donor – or otherwise assisted – conception, comparisons are often made with adoption and parental order certificates, which record a transfer of legal parenthood in the context of adoption and surrogacy, respectively. In both cases, it is obvious that the person’s certificate is not an original birth certificate, prompting any curious individual to follow the paper trail to their original entry; this, of course, records the birth parents or sometimes the birth mother alone. However, these parents may or may not be the child’s genetic parents, so it seems odd to make this comparison. Birth parents are recorded because they, unlike most^14^ gamete providers, are legally responsible for the child at birth. To record persons with no legal connection or responsibility for the child would be a very fundamental and significant change to the birth registration system, and is one that demands careful consideration in light of the wider normative context in which birth registration operates.

This leads to my third observation, which is that a normative narrative of family and kinship relations has always underpinned the birth registration system, and shaped its meaning in society. The information recorded by the state – however partial and prescriptive – is informed by the normative politics of family life. While the specifics of such may shift over time, the politics seem to remain tenaciously informed by gendered perceptions of the two-parent family model, in which children are deemed to have, at most, two ‘real’ parents (Fineman, 1995, McCandless and Sheldon, 2010). To name gamete providers on a birth certificate, without first unshackling birth registration from this normative family model, would, I think, place legal parents who do not share a genetic connection with their child in a precarious legal and sociocultural position. Permitting the registration of more than two legal parents where three parents or more are collaboratively raising the child may offer one way of challenging the normative impulses that underpin birth registration. Indeed, some jurisdictions have already introduced this possibility (e.g. British Columbia). Research examining the effects of such legal reforms would be extremely useful in allowing us to reach conclusions on whether it is possible to unshackle birth registration from the normative two-parent family model, or whether it simply re-emerges in different guises.

Either way, it is this normative underpinning which makes it crucial that any law reform project starts by asking and attempting to clarify the very basic question of the actual purpose of birth registration; only then can we determine what information is appropriate to record. In urging the Law Commission to consider this preliminary question, I do not preclude the possibility that we might seek to change the purpose of birth registration from what it was in historical terms, or charge it with multiple purposes. However, we do need to be realistic about what we ask of an administrative procedure such as birth registration, and what legal reform can achieve in light of the wider sociolegal context in which the system operates. In terms of legal context, we cannot consider the reform of birth registration law without also considering developments in other substantive areas of law, such as citizenship and family law.

In my fourth observation, I restrict my comments to developments in family law, where birth registration has become an increasingly important social policy tool in relation to parenting. Section 111 of the Adoption and Children Act 2002 reformed the law so that voluntary joint birth registration conferred automatic parental responsibility to unmarried fathers (Sheldon, 2009, Wallbank, 2009), rather than the father having to come to a parental responsibility agreement with the legal mother or apply to the court for a parental responsibility order (Children Act 1989, Section 4). The Welfare Reform Act 2009, although as yet unimplemented, makes legislative provision for the compulsory joint registration of all non-marital births, and amends the Children Act 1989 to automatically confer parental responsibility to unmarried fathers. With these recent amendments, birth registration is no longer regarded in family law as simply a written legal record or evidence of who a child’s parents were at the moment of birth, but rather, as a social policy tool that should facilitate the parent–child relationship through the conferment of various parental legal rights and entitlements (McCandless, 2011). Whether a paper certificate can ever successfully encourage parent–child relationships remains to be seen, and we should not forget that family law also prescribes who can be regarded as a legal parent and thus eligible to be named on a birth certificate. However, these changes do make us conscious of the role and power of committing something to paper, and the significance of what has been described as ‘textually mediated organisation’ by the state (Breckenridge and Szreter, 2012). This reminds us of the important role of registration, particularly of vital information such as births and deaths, in the politics of modern statecraft, and just as scientific ‘facts’ are never entirely objective,^15^ so the information recorded through processes of registration are never entirely ‘neutral’.

## Conclusion

In response to the Law Commission’s consultation question on whether it should review the law governing the registration of births, the answer is surely ‘yes’. Legal challenges to the current system are increasing, and while the context of each challenge varies, what we see in common across different contexts is a growing significance being placed on what individuals regard as subjectively ‘accurate’ information being recorded, and a desire to have significant life events ‘recognized’. These developments relate, in part, to people’s individual circumstances and family narratives not being countenanced in the standardized birth registration procedures and forms, but they also relate to the multiple, and sometimes competing and contradictory, understandings that people have of the purpose of birth registration. As birth certificates are increasingly required for identification, such challenges are likely to increase, particularly as different forms of parenting, family life and gender become increasingly visible and acceptable. While some may argue that legal reform in this area would not benefit a substantial enough number of people, such would only be true if the Law Commission was to cast the scope of the project in a narrow fashion. Underpinning some specific reform questions with a much wider consideration of the purpose of birth registration in the contemporary era would be an extremely useful project for wider society, with implications for everybody given the compulsory nature of birth registration. While there will undoubtedly be a huge number of proposals for the 13^th^ Programme,^16^ many of which will seem worthy of reform, how many will involve a compulsory procedure that has remained fairly stagnant since the early 19^th^ century, despite significant developments in family life; understandings of gender in society; and the information which the state holds on individuals in other administrative, statistical, biometric and healthcare databases?

There is significant – and really interesting – work to be done in this area. In legal terms, the past decade has seen a growing body of academic scholarship on birth registration, but, like reform, this has been piecemeal. There is plenty of scope for more sustained considerations, particularly those of an empirical and comparative nature. Placing birth registration law on the legal reform agenda would certainly highlight registration as a serious topic for academic and other research, and would lead to greater societal understanding of both the purpose and meaning of this increasingly important procedure and ritual in contemporary society.

## Footnotes

1 According to the Human Fertilisation and Embryology Authority’s most recent statistics, live birth rates in fertility clinics in 2013 were as follows: 589 following in-vitro fertilization (IVF) with donated sperm; 597 following IVF using donated eggs; and 590 following donor insemination (HFEA, 2014). The statistics do not detail the number of live births following donated embryo transfer, nor do they detail donor insemination births that take place in a non-clinical setting.

2 For example, in Torfaen County Council in Wales, residents applying for the nappy collection service as part of their fortnightly waste collection must provide a copy of their child(ren)’s birth certificate [http://www.torfaen.gov.uk/en/RubbishAndRecycling/Householdwaste-domesticbins/YellowBag/Yellow-Bag.aspx (last accessed 8 May 2017)].

3 Note that the Law Commission requires confirmation from a government department that it has a 'serious intention' to take law reform forward before it can include a project in its programme. At this stage, we can only speculate on whether the Law Commission have mooted a project on birth registration law in their 13^th^ Programme at the suggestion of a government department and which department it might be.

4 It therefore differs from previous legal cases, such as Joanna Rose’s judicial review case against the UK’s donor anonymity policy (*Rose v Secretary of State for Health and the HFEA* [2002] EWHC 1593), which more directly challenged state obligations relating to the management of personal data.

5 Examples beyond donor conception and legal parenthood include campaigns by intersex and transgender persons for more responsive gender recognition (Cooper and Renz, 2016: 484); campaigns for death certificates to be replaced by birth certificates following a stillbirth (Sanger, 2010); and campaigns for certificates registering the early pregnancy loss of a miscarriage (Pearlman and Foster, 2015).

6 I use this term to denote families whereby more than two persons ‘collaborate’ to have a child together, sometimes with the joint intention of raising the child together.

7 It is beyond the scope of this paper to discuss the contested relationship between sex and gender, beyond noting that birth registration in England and Wales records a person’s ‘sex’ as either ‘male’ or ‘female’. It is also beyond the scope of this paper to analyse the relationship of ‘sex’ registration to ‘gender re-assignment’ in the Gender Recognition Act 2004 (see further Cowan, 2005). On the possibility of eliminating the role of state law in determining and assigning a person’s sex/gender, see Cooper and Renz (2016).

8 The systems in Scotland and Northern Ireland are similar but separate.

9 The following people can act as an informant: mother; father; occupier of the house where a child is born; any person present at the birth; and a person having charge of the child.

10 Note that while a father’s name and occupation is recorded on a marriage certificate in England and Wales, no details are recorded for a bride or groom’s mother. This was the subject of a recent campaign directed at Members of Parliament (https://www.change.org/p/mothers-names-should-be-on-marriage-certificates).

11 My thanks to an anonymous reviewer for highlighting this important point.

12 For example, if you were born in the UK on or after 1 January 1983, you will need your long birth certificate in order to apply for a first adult passport. If you were born before this date, your short birth certificate will suffice.

13 Although it would, of course, be interesting to research how many parents do actually re-register their child’s birth. My anecdotal research with family and friends would indicate that very few do so.

14 It is important to acknowledge that some gamete providers may well intend to take a more active role in a child’s life, to include co-parenting arrangements.

15 For a discussion on the impossibility of ‘fixing’ a definition of genetic parenthood, particularly in light of new technologies such as MitDNA transfer, see McCandless and Sheldon (2014).

16 For 12th Programme, there were 250 suggested areas during the consultation, leading to nine new projects.
